# Scratch-Induced Deformation Behavior of Wire-Arc Directed Energy Deposited α-Titanium

**DOI:** 10.3390/ma18030724

**Published:** 2025-02-06

**Authors:** Blanca Palacios, Sohail M. A. K. Mohammed, Tanaji Paul, Gia Garino, Carlos Maribona, Sean Langan, Arvind Agarwal

**Affiliations:** 1 Cold Spray and Rapid Advanced Deposition Laboratory, Department of Mechanical and Materials Engineering, Florida International University, 10555 West Flagler Street, Miami, FL 33174, USA; bpala021@fiu.edu (B.P.); smohamme@fiu.edu (S.M.A.K.M.); tpaul@fiu.edu (T.P.); ggari005@fiu.edu (G.G.); 2 Department of Materials Science and Engineering, Cornell University, Ithaca, NY 14850, USA; cam487@cornell.edu; 3 Solvus Global LLC, 104 Prescott Street, Worcester, MA 01605, USA; sean.langan@solvusglobal.com

**Keywords:** wire-arc directed energy deposition, scratch response, plastic deformation, electron back scattered diffraction (EBSD), titanium

## Abstract

This study investigates the scratch response of α-phase commercially pure titanium (cp-Ti) produced via wire arc directed energy deposition (WDED), focusing on the thermal history and directional effects. Progressive scratch tests (1–50 N) revealed heterogeneous wear properties between the top and bottom layers, with the top layer exhibiting higher material recovery (58 ± 5%) and wear volume (5.02 × 10^−3^ mm^3^) compared to the bottom layer (42 ± 5% recovery, 4.46 × 10^−3^ mm^3^), attributed to slower cooling rates and coarser grains enhancing ductility. The variation in the properties stems from the thermal gradient generated during WDED. Electron backscatter diffraction analysis showed higher kernel average misorientation (KAM) in the bottom layer (0.84° ± 0.49° vs. 0.51° ± 0.44°), affecting plasticity by reducing dislocation and twin boundary mobility. No significant differences were observed between longitudinal and transverse orientations, with coefficients of friction averaging 0.80 ± 0.12 and 0.79 ± 0.13, respectively. Abrasive wear dominated as the primary mechanism, accompanied by subsurface plastic deformation. These findings highlight the significant influence of WDED thermal history in governing scratch resistance and deformation behavior, providing valuable insights for optimizing cp-Ti components for high-performance applications.

## 1. Introduction

Ti and its alloys are highly favorable materials to produce structural components in marine applications, such as propellers, power generation devices, hull structures, seawater desalination devices and other ship parts, due to their high strength-to-weight ratio and excellent electrochemical stability [1,2,3]. Among these materials, commercially pure titanium (cp-Ti) is particularly suited for such applications because of its specific strength (~340 MPa), low density (~4.5 g/cm^3^), and superior corrosion resistance [4], which makes it ideal for ensuring long-term durability in harsh marine environments. Traditionally, cp-Ti components have been manufactured using conventional methods like casting [5,6]. While casting is effective for producing components in large quantities and with diverse geometries, it often falls short when creating intricate designs or complex shapes required in advanced applications.

In recent years, wire arc-directed energy deposition (WDED) has emerged as a promising additive manufacturing technology for producing medium-to-large-scale titanium components. This process offers significant advantages over traditional casting, including greater design flexibility, reduced material waste, and higher energy efficiency [7,8]. However, WDED’s unique thermal cycles and variable deposition paths can introduce microstructural inhomogeneity and anisotropy, potentially affecting the material’s mechanical performance, including its scratch behavior. Understanding these effects is critical to improving the reliability of WDED cp-Ti components in demanding environments. Cp-Ti often possesses poor tribological performance, characterized by low surface hardness, high coefficients of friction (COF), and susceptibility to adhesive and abrasive wear [9]. Abrasive wear is typically followed by adhesion and transfer layer formation during sliding interactions, where material is transferred between surfaces. Disruption of the protective oxide layer during contact further exacerbates wear by exposing reactive surfaces [10]. This phenomenon may be particularly relevant for as-deposited cp-Ti produced via WDED under inert environmental conditions, where limited oxygen diffusion influences the formation of the oxide layer and, consequently, the scratch behavior of the surface. For instance, Chen et al. [3] reported the scratch and wear response of as-received and heat-treated cp-Ti Grade 2 rolled samples, noting that the as-received samples underwent severe plastic deformation, causing surface smearing due to the thin oxide layer. Similarly, studies have highlighted that the wear mechanisms in cp-Ti are closely linked to its microstructural features, such as grain size and the interaction of wear-induced deformation with the material’s crystalline structure [11,12,13,14]. In addition, the research on real-time imaging of additive manufacturing processes further assisted in correlating these properties with the manufacturing process [15,16,17]. However, the effects of WDED thermal cycles and deposition directionality on the scratch-induced mechanisms remain largely unexplored.

This study addresses this gap by investigating the scratch response of single α-phase cp-Ti produced by WDED. To achieve this, experiments were strategically conducted at different locations within the bulk material, in varying orientations, and across distinct layers along the buildup direction to evaluate the combined effects of deposition directionality and thermal history. Specifically, the relationship between deposition patterns, thermal gradients, scratch resistance and plastic deformation at progressively applied loads is examined. Material recovery, penetration depth, frictional response, and microstructural deformation near the scratch groove are analyzed to provide a comprehensive understanding of the material’s behavior. These findings offer fundamental insights into the scratch-induced wear mechanisms of WDED cp-Ti, which will inform strategies for tailoring surface properties to enhance the durability of marine components, further advancing the potential of additive manufacturing.

## 2. Materials and Methods

### 2.1. Wire Arc Directed Energy Deposited Material

**Figure 1:**
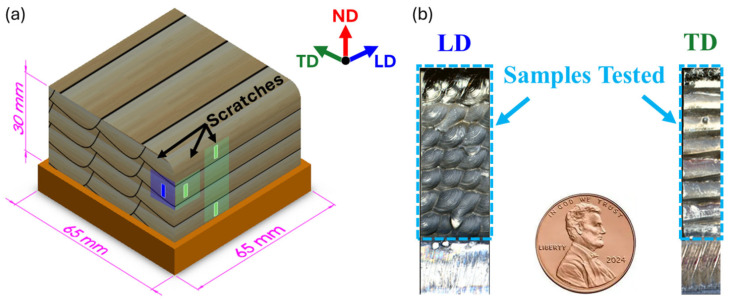
(**a**) Schematic of the WDED-deposited cp-Ti component (65 mm × 65 mm × 30 mm) showing longitudinal (LD), transverse (TD), and normal (ND) directions. (**b**) Machined samples for scratch testing, with the LD sample showing welding bead patterns and the TD sample displaying layered buildup.

The WDED process was conducted using 1 mm diameter commercially pure titanium (cp-Ti) wire, classified as ASTM Grade 2. Eight vertical layers were deposited, each containing 16 parallel tracks of solidified cp-Ti, in an inert argon (Ar) atmosphere to prevent oxidation. Following a continuous raster pattern, the process produced a bulk component with final dimensions of 65 mm × 65 mm × 30 mm, as presented in Figure 1a. For this study, the longitudinal direction (LD) aligns with the arc’s travel direction, the transverse direction (TD) is perpendicular to the arc movement, and the normal direction (ND) represents the build direction, as indicated by the axis system. The machined samples prepared for scratch testing are shown in Figure 1b. The LD sample exhibits the pattern of multiple welding beads, while the TD sample displays superimposed layers along the buildup direction. These images represent the samples in their as-received condition for reference.

### 2.2. Sample Preparation

Metallographic preparation of the specimens was performed by machining the bulk deposit using a high-speed diamond saw (TechCut 5X^TM^, Allied HighTech Products, Compton, CA, USA) at 1000 rpm and a linear speed of 0.05 in/min to minimize surface deformation from cold working. Grinding was conducted manually using silicon carbide (SiC) papers with grit sizes of 400 (22 µm) and 600 (15 µm) in accordance with ANSI standards. Final polishing involved a chemical–mechanical approach using a mixture of 0.05 µm amorphous colloidal silica suspension, hydrogen peroxide (H_2_O_2_, 30 wt.%), and commercially available Kroll’s reagent (70, 22, and 8%, respectively). This polishing was performed on a porous, no-nap, chemical-resistant pad, optimizing the mixture’s effectiveness for high material removal rates while achieving a deformation-free, mirror-like surface finish. Residual particles were removed via ultrasonic cleaning in methanol for 30 min. Etching was not performed on the samples, as the hexagonal close-packed (HCP) crystal structure’s interaction with polarized light enabled effective characterization of the microstructure near the scratch grooves.

### 2.3. Experimental Setup for Scratch Experiments

Scratch tests were performed on a micro-scratch tester (Revtest RST3, Anton Paar, Inc., Houston, TX, USA) equipped with a Rockwell indenter featuring a tip radius of 50 mm. The scratch tests were conducted under progressive loading conditions with the applied load ranging from 1 to 50 N at a speed of 1.5 mm/min over a scratch length of 3 mm. Data on acoustic emission (AE), COF, instantaneous penetration depth (h_inst_), and residual depth (h_true_) were collected during testing, while an integrated optical microscope provided panoramic images of the scratch track. AE and COE range and average were calculated for comparison purposes. Material recovery percentage was calculated at every recorded data point from the scratch test, providing the range of variation. Averages and standard deviations were also calculated for comparison purposes. Scratch-induced properties were analyzed and correlated with wear and deformation mechanisms in the microstructure. Scratches were performed in layer 4 of the deposited block, approximately 14 mm from the substrate, in both LD and TD samples to investigate possible anisotropy behavior based on the WDED deposition directionality. Additional scratches were conducted in the bottom and top layers of the TD sample along the buildup direction to evaluate the effect of the WDED-induced thermal gradient on the scratch response of the cp-Ti material. A representative schematic of the scratch test methodology is illustrated in Figure 1a.

### 2.4. Scratch-Induced Microstructural Analysis by Imaging and Diffraction Techniques

The analysis of the scratch paths was conducted using integrated diffraction and microscopy techniques. An optical microscope (Axioscope 5, Zeiss Microscopy, DE) was employed, combining polarized light and differential interference contrast (DIC) methods to capture low-magnification, individual and stitched images of the full scratch path. Higher magnification images were captured employing a field-emission scanning electron microscope (FESEM, JEOL-F100, JEOL Ltd., Akishima, Tokyo, Japan) to provide insights into the wear and deformation mechanisms produced during scratching. The scratch direction is from left to right in all the images presented in this study.

**Figure 2:**
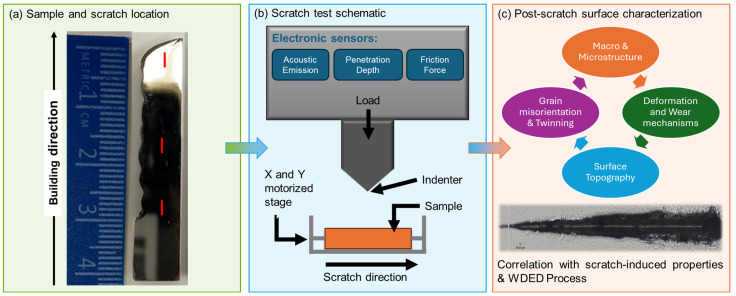
(**a**) Representative schematic of the WDED cp-Ti as-polished sample showing scratch locations before testing. (**b**) Outline of a scratch tester, (**c**) Schematic of the post-scratch test characterization employing a combination of imaging techniques to obtain the microstructural features near the scratch and their correlation to the material’s scratch response.

Structural features near the scratches, including crystallographic orientation and deformation, were examined using electron backscatter diffraction (EBSD) on the SEM equipped with an EBSD detector (Symmetry S2, Oxford Instruments, Abingdon, UK). EBSD scans were conducted at an accelerating voltage of 20 kV, with the specimen stage tilted to 70°, acquiring Kikuchi patterns at a step size of 1.5 µm. Grain orientation, boundaries and Kernel average misorientation (KAM) mapping were post-processed using Oxford’s Aztec Crystal 2.2 software. KAM analysis used a 5 × 5 square kernel and a maximum misorientation angle of 5° to calculate and visualize local lattice misorientations within the cp-Ti HCP phase. Wear track profiles of the scratched samples were evaluated with optical profilometry (Nanovea, Irvine, CA, USA). Calculations of wear volume from top-view profilometry images were performed using Mountains Lab Premium 10 software (Digital Surf, Besancon, France), ensuring precise analysis of the scratch geometry. A summary schematic of the experimental method utilized to obtain the current study data, as described in Section 2, is provided in Figure 2a–c as follows:

## 3. Results and Discussions

### 3.1. Influence of Deposition Direction on Wear Mechanisms and Deformation Behavior

**Figure 3:**
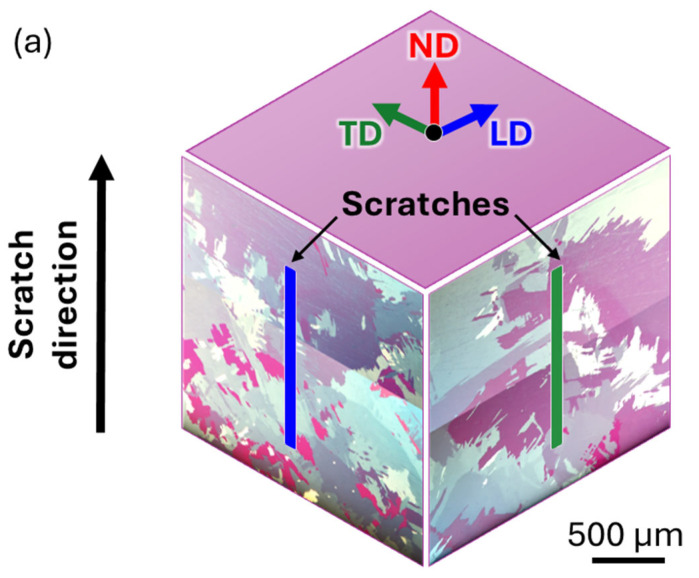

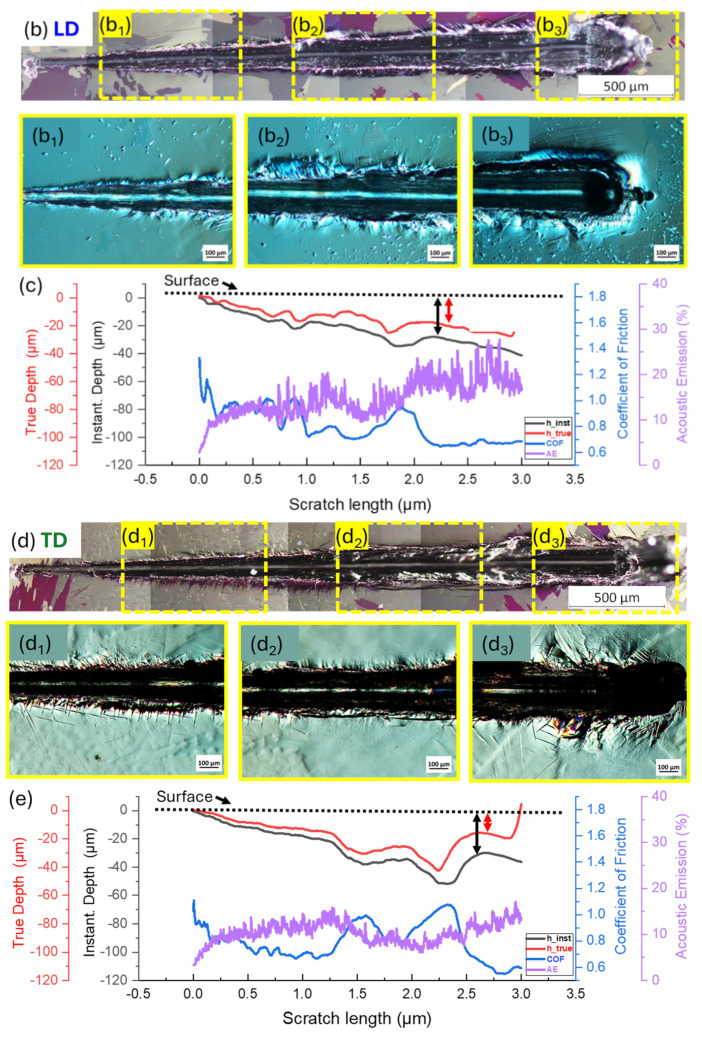
(**a**) Representative WDED cp-Ti polarized micrographs of LD and TD microstructure. Scratch track polarized micrographs ((**b**) for LD; (**d**) for TD). DIC micrographs of 3 different regions revealing twinning and material pile-ups near the scratch flanks ((**b_1_**–**b_3_**) for LD; (**d_1_**–**d_3_**) for TD). (**c**,**e**) Scratch-induced properties profiles: true depth of penetration (h_true_), instantaneous depth of penetration (h_inst_), coefficient of friction (COF), and acoustic emissions (AE). Material recovery is marked with black and red double tip arrows.

The role of deposition pattern on the microstructure of WDED components is crucial as it influences wear behavior and deformation mechanisms. In the present study, scratch tests were conducted in both LD and TD directions to explore any potential anisotropic effects arising from the deposition pattern. The microstructural characteristics in both directions (LD and TD) exhibit comparable grain sizes and morphologies, as seen in the stitched microstructure presented in Figure 3a. The WDED process results in a heterogeneous microstructure due to the large melt pool size and relatively slow cooling rates [18]. This leads to the formation of coarse grains with a size of approximately 1 mm. These grains are interspersed with nested fine grains of around 100 μm, creating a complex structure that may contribute to anisotropy during deformation due to heterogeneity. These microstructural features are significant in determining the material’s response to directional stresses.

The polarized micrographs of the complete scratch performed in both directions are presented in Figure 3b,d for LD and TD, respectively. Severe plastic deformation of the microstructure near the groove is evident even at the lowest applied loads during the progressive (1–50 N) scratches in both the LD and TD samples, as shown in optical micrographs using DIC in Figure 3(b_1_–b_3_) for LD, and Figure 3(d_1_–d_3_) for TD. These micrographs reveal an avalanche of twinning structures, characterized by their thin lamellae morphology, forming along the flanks of the scratch path. These twins are accompanied by material removal in the form of pile-ups that contribute to plastic deformation. The initiation of twinning at the lowest loads suggests that the material’s response is highly sensitive to shear stress, which facilitates twinning as an alternative deformation mechanism to slip [19,20,21]. This behavior is attributed to the HCP crystal structure of cp-Ti, which has low symmetry and a limited number of independent slip systems, requiring the activation of twinning to accommodate plastic strain [20,22,23].

During the nucleation of twins, thin lamellae form at a velocity comparable to the speed of sound and thicken as stress increases due to the constant motion of the twin boundary. This rapid process generates acoustic waves, commonly referred to as the “twin cry” effect [23], which can be recorded. The AE signal (purple profiles) in Figure 3c,e averages 14.9 ± 3.7% for LD and 10.4 ± 1.9% for TD, exhibiting multiple spikes throughout the test. These spikes are indicative of sudden energy releases, primarily associated with deformation mechanisms such as twin nucleation and propagation. Twin nucleation involves a localized reorientation of the crystal lattice, which necessitates the overcoming of an energy barrier to rearrange atomic planes into a new orientation. The energy released during this process stems from the sudden relaxation of elastic strain energy stored in the lattice as the material transitions into the twinned state [22,24,25]. This release is accompanied by localized stress redistribution, which generates the AEs captured as spikes. The frequency and amplitude of the AE spikes provide evidence of the continuous and progressive activation of twinning mechanisms along the scratch path. Higher frequencies correspond to repeated twin nucleation events, while larger amplitudes may indicate significant energy releases during extensive twin propagation. Together, these observations highlight the material’s intrinsic response to directional wear and deformation.

COF evaluates the qualitative analysis of the wear mechanism. The COFs in the LD and TD directions averaged 0.80 ± 0.12 and 0.79 ± 0.13, respectively, indicating no significant difference in the sliding behavior of the contact surfaces in the two tested directions. This average COF is slightly higher than the typical value for wrought cp-Ti, which is ~0.64 ± 0.06 [26], potentially due to the dynamic interaction between the transfer layer of the WDED material and the counterpart leading to friction fluctuations [27,28]. The COF displayed oscillatory behavior at different scratch lengths and values ranging between 0.64–1.08 for the LD and 0.55–1.07 for the TD, see blue profiles in Figure 3c,e. The oscillations are consistent with the “stick-slip” effect, often attributed to the localized fracture of the transfer layer and particle interactions at the sliding interface, as reported in prior studies [26,29,30]. No direct correlation was found between the COF fluctuations and the applied load, suggesting that these variations are primarily related to the inherent response of the material against frictional forces.

In addition, fluctuations in the penetration depth align with the oscillations in COF, with drop-downs in-depth occurring at points of COF variation. This suggests a coupling between frictional response and deformation, where changes in surface interaction affect both the sliding resistance and depth of penetration. The depth of penetration increases with the applied load, as expected. This increase in depth is due to the material’s response to progressively higher stress, which results in increased plastic deformation. The scratch test reveals a distinction between the h_inst_, recorded while the load is applied (black profiles in Figure 3c,e), and the h_true_, measured after load removal (red profiles in Figure 3c,e). This difference is due to the elastic properties of cp-Ti, which allows partial recovery of the material after load removal, resulting in a h_true_ that is shallower than h_inst_. To quantify this recovery behavior, material recovery was calculated using the equation:

(1) $$Material\;recovery\;\left(\%\right)=\frac{h_{inst}-h_{true}}{h_{inst}}\times \;100,$$

The calculated average material recovery is estimated as 35 ± 8% for LD and 30 ± 10% for TD, showing a similar response between the directions tested. The insignificant deviation suggests that the elastic recovery behavior of WDED cp-Ti is nearly isotropic under the conditions tested (scratches at the same deposited layer at ≈ 14 mm from the substrate), with both directions displaying similar resilience before transitioning to permanent deformation. A summary of the scratch-induced properties in LD and TD is presented in Table 1.

**Figure 4:**
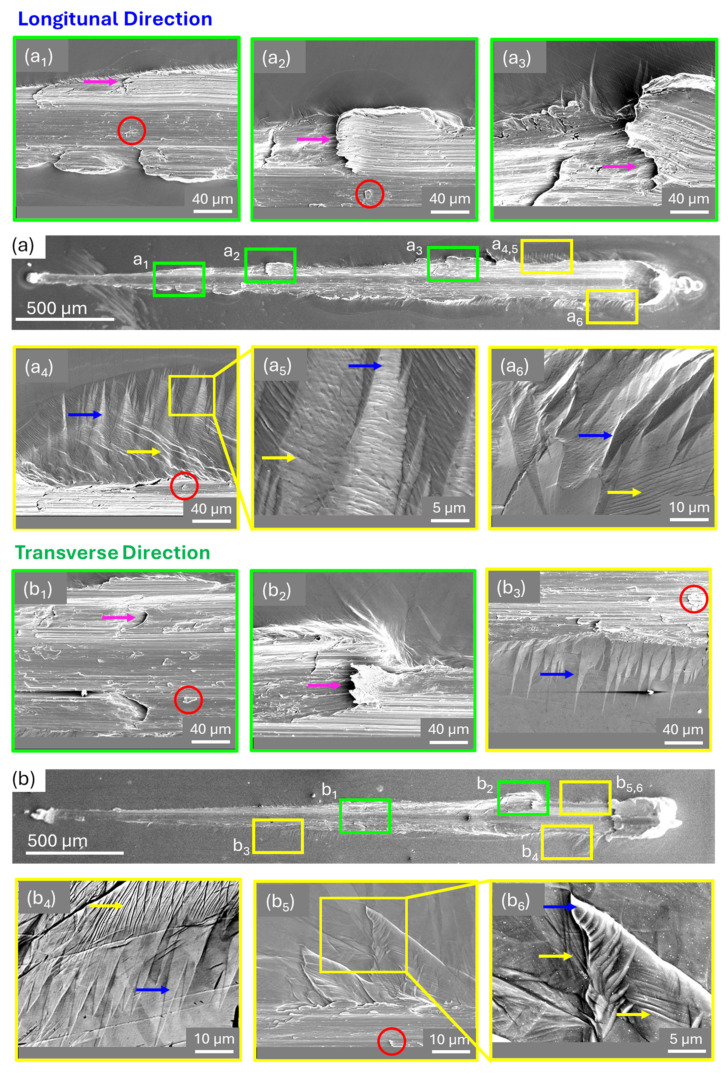
SEM images showing complete scratch morphology and regions of scratch-induced fluctuations in (**a**) LD and (**b**) TD directions. ((**a_1_**–**a_3_**) for LD; (**b_1_**,**b_2_**) for TD) Green boxes highlight delamination cracks (pink arrows) and material accumulation (red circles) within the groove. ((**a_4_**–**a_6_**) for LD; (**b_3_**–**b_6_**) for TD) Yellow boxes show subsurface plastic deformation, quilted-looking twin structures (blue arrows), and slip lines (yellow arrows) confirming deformation mechanisms observed in optical micrographs.

Regions exhibiting significant data fluctuations in the scratch-induced properties profiles at different scratch lengths were selected for SEM observation further to investigate the behavior along both LD and TD. SEM images in Figure 4a,b show the complete scratch morphology of LD and TD, respectively. Higher-magnification views were taken from regions where the COF and penetration depths exhibited simultaneous peaks and drop-downs, respectively. For the LD, these phenomena were evident at approximately 0.64 mm, 0.88 mm, and 1.90 mm along the scratch path, and the corresponding high-magnification images of these regions are shown in Figure 4(a_1_–a_3_), highlighted in green boxes. Similarly, along TD, these occurrences were identified at approximately 1.57 mm and 2.28 mm, with high-magnification images of these regions shown in Figure 4(b_1_,b_2_), also marked with green boxes. The material shows evidence of subsurface shear stress leading to the formation of delamination cracks [26,27] in both LD and TD directions. These cracks grow parallel to the surface, eventually detaching material and leaving behind darker, irregular marks within the groove and along the flanks of the scratch (pink arrows in Figure 4). The morphology of the wear tracks also reveals plowing lines throughout the groove, indicative of severe abrasive wear as the primary wear mechanism across all load ranges. This behavior aligns with findings in the literature [26], which indicate that dry contact conditions often involve multiple simultaneous wear mechanisms.

Further evidence of subsurface plastic deformation is observed in the form of material accumulation within the groove (red circles in Figure 4) and a noticeable amount of deformation twins near the scratch (blue arrows in Figure 4), as highlighted in the yellow boxes, Figure 4(a_4_–a_6_) for LD and Figure 4(b_3_–b_6_) for TD. Twin lamellae structures, previously identified in optical micrographs (Figure 3), are also visible under SEM observation. These are characterized by the sharp contrast between the twins and the matrix [31,32], marked with the blue arrows within the yellow boxes in Figure 4. Slip lines, marked with yellow arrows in Figure 4, further highlight the material’s plastic deformation mechanisms during scratching. The twin structures exhibit a quilted-looking morphology [33,34], consisting of twins arrested at other twin boundaries and intersecting slip bands, consistent with deformation in HCP materials like cp-Ti. These twins form twin–twin junctions [19,33], where twin variants [35] interact under the stresses induced by scratching, as shown in Figure 4(b_5_,b_6_). Twin–twin junctions can significantly influence plastic deformation by redirecting slip and inducing localized hardening [20,21,36]. In the present study, evidence of slip band transmitting across twin boundaries is observed, as shown in Figure 4(a_4_,a_5_,b_4_,b_6_), indicating low resistance to slip transmission at the twin boundaries [37,38]. These findings demonstrate that the interaction of twins plays a crucial role in accommodating deformation and balancing strain energy under high shear stresses induced in the cp-Ti WDED during scratching at progressively increasing loads. However, no dependency on deposition directionality was observed for the occurrence of these mechanisms.

### 3.2. Heterogeneous Wear and Plasticity Response Induced by WDED Thermal Gradient

**Figure 5:**
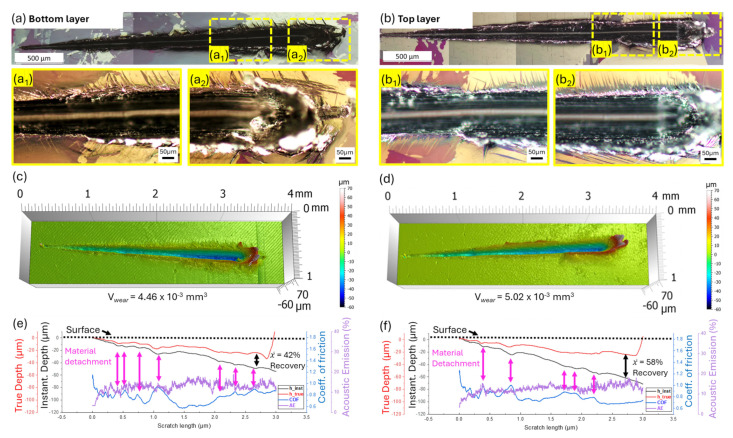
Polarized micrographs of bottom and top layer scratches of the WDED cp-Ti buildup ((**a**) for bottom layer; (**b**) for top layer). Higher magnification polarized micrographs show severe plastic deformation, material accumulation, and variations in twin distribution ((**a_1_**,**a_2_**) bottom layer; (**b_1_**,**b_2_**) top layer). Optical profilometry of scratch tracks, highlighting differences in penetration depth and pile-up height ((**c**) for bottom layer; (**d**) for top layer). Profiles of scratch-induced properties, COF, and AE%, indicating material detachment (pink arrows) and recovery differences (black arrows) influenced by thermal history ((**e**) for bottom layer; (**f**) for top layer).

Scratch tests were conducted on the bottom and top layers to evaluate the influence of the ΔT_WDED_ on the scratch response of cp-Ti. The complete morphology of the scratches is presented by the polarized optical micrographs in Figure 5a,b for the bottom and top layers, respectively. Severe plastic deformation in the form of material accumulation and multiple twin structures is evident in both layers. The twins exhibit quilted-looking structures [33,34] similar to those described in Figure 4. Variation is observed in the twin distribution, with the bottom layer showing a more inter-spaced twin arrangement compared to the more homogeneous deformation in the top layer at similar load ranges (see high-magnification micrographs in Figure 5(a_1_,a_2_,b_1_,b_2_)). This difference suggests anisotropic plastic responses in the subsurface, potentially leading to variations in twin–twin interactions and dislocation transmission mechanism at the twin boundary [19,33,38].

The optical profilometry results, presented in Figure 5c,d, provide a detailed assessment of the scratches. Profilometry was used to measure the maximum penetration depth and pile-up height at the highest applied loads (≈48 N). The maximum penetration depth was approximately 33.3 µm for the bottom layer and 25.5 µm for the top layer, while the maximum pile-up heights were 73 µm and 63 µm, respectively. These measurements show consistent trends and reveal significant heterogeneous behavior. To further evaluate wear performance, an approximate area of 1 mm^2^ framing the grooves was analyzed to calculate the wear volume (V_wear_). The V_wear_ was estimated to be 4.46 × 10^−3^ mm^3^ for the bottom layer and 5.02 × 10^−3^ mm^3^ for the top layer, corresponding to a ≈ 12% higher V_wear_ in the top layer. This difference can be attributed to the possible softening effect in the top layer due to the thermal conditions during the WDED. The observed softening in the top layer can be explained by heat accumulation during WDED [39,40]. As subsequent layers are deposited, reduced heat conduction into the substrate and increased heat retention in the bulk material results in slower cooling rates for the top layer [18,41]. This slower cooling may produce coarser grains and reduce dislocation density, leading to lower hardness and higher ductility [42]. Consequently, the top layer accommodates plastic deformation more uniformly, resulting in fewer pile-ups and more continuous deformation of the cp-Ti, as demonstrated in these results.

The profiles evaluated from scratch tests are presented in Figure 5e,f for the bottom and top layers, respectively, with a summary of calculated averages and ranges provided in Table 2. Notably, under stress, the indenter penetrates deeper into the top layer, resulting in a maximum h_inst_ of 68.4 µm, which is 35% higher than the maximum h_inst_ of 50.7 µm observed in the bottom layer under the same normal force of 48 N. Upon load release, however, the Δh (difference between h_inst_ and h_true_) is larger for the top layer. This suggests a difference in plasticity response, as reflected in the calculated average material recovery. Thus, the material recovery in the top layer was 58 ± 5%, approximately 16% higher than 42 ± 5% observed in the bottom layer scratch, further supporting the anisotropic behavior.

Similarly to the findings described in Section 3.1, the ranges and averages of COF and AE% are consistent across both layers (see Table 2), indicating that the wear mechanisms are primarily driven by the inherent material properties under frictional forces, with no significant relationship to WDED thermal effects. The COF profiles in Figure 5e,f exhibit fluctuations, with values deviating significantly from the averages, marked on the profile with the pink double arrows. This phenomenon is associated with material detachment failures in the form of delamination, as previously described in Figure 4.

**Figure 6:**
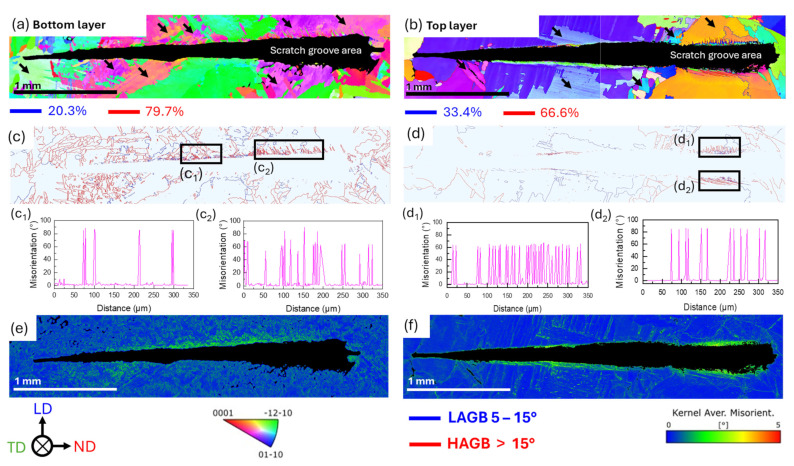
EBSD of scratches in the bottom (**a**,**c**,**e**) and top (**b**,**d**,**f**) layers. Grain orientation maps (**a**,**b**) show grain structure and misorientation (Black region in the scratch groove corresponds to the EBSD zero solutions due to severe plastic deformation and black arrows mark the grains with misorientation gradients). Twin lamellae and misorientation angles (**c**,**d**) highlight deformation mechanisms. Misorientation peaks plots (**c_1_**–**c_3_**,**d_1_**–**d_3_**) near the scratch flanks. KAM maps (**e**,**f**) showing differences in misorientation angle distribution within the grain of the WDED cp-Ti.

To elucidate the degree of deformation and misorientations in the microstructure, EBSD was used in the vicinity of the scratch, providing detailed information on scratch-induced plastic deformation mechanisms. The EBSD orientation maps reveal a microstructure with randomly oriented grains in both the top and bottom layers, as presented in Figure 6a,b. However, a few grains, as marked with a black arrow, exhibit a gradient color, indicating intragranular misorientation attributed to varying solidification rates during the WDED process.

With the increase in the load of scratching due to progressive load, the twins evolved as captured from the orientation maps in Figure 6a,b, supporting the findings presented in Figure 5. These twins appear larger and thicker under increased load. Grains near the scratch groove reorient due to the stresses imposed by the indenter, with twins adjacent to the groove displaying varying misorientation angles. Regions where twins were clearly identified were selected for analysis, marked with black boxes in Figure 6c,d, highlighting distinct twin morphologies. For the bottom layer, Figure 6(c_1_) shows twin misorientation angles of 85.2° ± 0.6°, corresponding to {$10\bar{1}2$} <$\bar{1}011$> type tension twins. Figure 6(c_2_) reveals multiple twin variants within the same parent grain (similar to those reported in [43]), with misorientation values of 51.2° ± 3° and 63.8° ± 3°, corresponding to {$10\bar{1}0$}<$\bar{1}012$> and {$11\bar{2}2$}<$\bar{11}23$> type compression twins, along with misorientation values of 85.2° ± 0.6° for {$10\bar{1}2$} <$\bar{1}011$> type tension twin. In the top layer, Figure 6(d_1_) shows misorientation values of 62.6°± 2° for {$11\bar{2}2$}<$\bar{11}23$> type compression twin, while Figure 6(d_2_) indicates misorientation values of 85.2° ± 0.9° for {$10\bar{1}2$} <$\bar{1}011$> type tension twins. These findings suggest that twin nucleation in the top layer occurs more homogeneously. In contrast, the non-uniform twin nucleation in the bottom layer is attributed to the greater impact of the thermal strain in the first deposited layers [44], where intragranular misorientation is more evident within the microstructure. This results in a more complex grain reorientation during scratching, as the relationship between the scratch travel direction and the misorientation in the parent grain strongly influences twin formation.

The level of stress induced in the grains adjacent to the scratch is analyzed using Kernel average misorientation (KAM) maps in the bottom and top layers (Figure 6e,f, respectively). The average KAM value was higher for the bottom layer (0.84° ± 0.49°) compared to the top layer (0.51° ± 0.44°), reflecting greater intragranular misorientation in the bottom layer. Especially, at the ridges of the scratch, the misorientation was found to be the highest. To distinguish this, the fraction of deformation-free regions with a threshold of 1° was established, defining the area fraction (Area_KAM_ ≤ 1°) as free of significant deformation. The bottom layer showed a lower Area_KAM_ ≤ 1 ° (74.5%) compared to the top layer (91.1%), indicating a more widespread and heterogeneous misorientation distribution within the grains in the bottom layer. This fact may potentially reduce the mean free path for dislocation and twin boundary motion. The lower mean free path impacts the formation of scratch-induced deformation mechanisms, such as twin nucleation and thickening, which appear more dispersed in the bottom layer. The higher obstacle density faced by twin boundaries in the bottom layer likely limits their ability to nucleate and propagate efficiently [45,46]. KAM maps also reveal widespread strain regions adjacent to the scratch grooves, attributed to localized scratch-induced deformation. These findings correlate with the observed plasticity responses during scratch tests, where the top layer exhibited more ductile behavior, higher material recovery, and fewer pile-ups compared to the bottom layer. This highlights the critical influence of thermal history on shaping deformation mechanisms, dislocation activity, and the scratch response in WDED cp-Ti.

## 4. Conclusions

This study comprehensively examined the scratch response of α-phase commercially pure titanium (cp-Ti) manufactured using wire arc directed energy deposition (WDED), focusing on the effects of deposition direction and thermal gradients. The findings indicate that directional anisotropy (LD vs. TD) had minimal impact on wear performance, with coefficients of friction and material recovery showing comparable values (35 ± 8% in LD and 30 ± 10% in TD). However, significant differences were observed between the top and bottom layers of the build, attributed to thermal gradients along the build direction. The top layer demonstrated higher material recovery (58 ± 5%) and wear volume (5.02 × 10^−3^ mm^3^) compared to the bottom layer (42 ± 5% material recovery, 4.46 × 10^−3^ mm^3^), attributed to slower cooling rates and coarser grain structures which enhances ductility. Similarly, EBSD analysis revealed higher kernel average misorientation (KAM) values in the bottom layer (0.84° ± 0.49° vs. 0.51° ± 0.44° in the top layer), which reduced the mean free path for dislocation and twin boundary mobility. This contributed to lower plasticity in the bottom layer and affected its deformation response. These differences underscore the role of thermal gradients in shaping grain boundary distribution, dislocation density, and deformation mechanisms, ultimately influencing wear behavior. Thus, the study highlights the importance of understanding thermal history effects in additive manufacturing to optimize the mechanical performance and scratch resistance of components. These insights provide a foundation for designing more durable and reliable titanium components for applications involving complex loading and frictional forces.

## Figures and Tables

**Table 1:** Scratch-induced properties comparison based on the orientation of the WDED cp-Ti.

Scratch Property Description		Orientation	
		Longitudinal	Transverse
Material Recovery (%)	Average	35 ± 8	30 ± 10
	Range	19 − 52	16 − 53
COF	Average	0.80 ± 0.12	0.79 ± 0.13
	Range	0.64 − 1.08	0.55 − 1.07
AE (%)	Average	14.9 ± 3.7	10.4 ± 1.9
	Range	8.4 − 27.8	6.0 − 15.8

**Table 2:** Scratch-induced properties comparison based on the location of the WDED cp-Ti buildup.

Scratch Property Description		Orientation	
		Bottom Layer	Top Layer
Max. h_inst_ (µm) at Crit. Load (N)	Value	50.7 at 48.5	68.4 at 48.7
Max. h_true_ (µm) at Crit. Load (N)	Value	33.3 at 47.4	25.5 at 47.7
Material Recovery (%)	Average	42 ± 5	58 ± 5
	Range	29 − 62	47 − 72
COF	Average	0.76 ± 0.09	0.79 ± 0.08
	Range	0.57 − 0.95	0.67 − 1.01
AE (%)	Average	12.9 ± 1.8	12.8 ± 1.7
	Range	7.2 − 18.7	6.6 − 18.3
V_wear_ (mm^3^)	Value	4.46 × 10^−3^	5.02 × 10^−3^

## Data Availability

The original contributions presented in this study are included in the article. Further inquiries can be directed to the corresponding author.
